# Dataset on the comparative proteomic profiling of mouse saliva and serum from wild type versus the dystrophic *mdx-4cv* mouse model of dystrophinopathy

**DOI:** 10.1016/j.dib.2018.10.082

**Published:** 2018-10-27

**Authors:** Sandra Murphy, Margit Zweyer, Rustam R. Mundegar, Dieter Swandulla, Kay Ohlendieck

**Affiliations:** a Department of Biology, Maynooth University, National University of Ireland, Maynooth, Co. Kildare, Ireland; b Institute of Physiology II, University of Bonn, D‑53115 Bonn, Germany

## Abstract

The comparative proteomic data presented in this article provide supporting information to the related research article "Proteomic identification of elevated saliva kallikrein levels in the *mdx-4cv* mouse model of Duchenne muscular dystrophy " (Murphy et al., 2018). Here we provide additional datasets on the comparative proteomic analysis of saliva and serum proteins and the mass spectrometric identification of kallikrein isoform Klk-1 in wild type versus *mdx-4cv* saliva specimens. The data article presents the systematic identification of the assessable saliva proteome and the differential presence of proteins in saliva versus serum samples. Representative mass spectrometric scans of unique peptides that were employed to identify the kallikrein isoform Klk-1 in wild type versus *mdx-4cv* saliva specimens are provided. The dataset contains typical saliva-associated marker proteins, including alpha-amylase and albumin, as well as distinct isoforms of cystatin, serpin, kallikrein, cathepsin, glutathione transferase, carbonic anhydrase, mucin, pyruvate kinase, and aldolase.

**Specifications table**

**Table:** 

Subject area	Biology
More specific subject area	Biomedicine
Type of data	Tables, MS/MS scans, Venn diagram
How data was acquired	LC-MS/MS analysis, using an Ultimate 3000 NanoLC system (Dionex Corporation, Sunnyvale, CA, USA) coupled to a Q-Exactive mass spectrometer (Thermo Fisher Scientific)
Data format	Analyzed
Experimental factors	Protein was extracted from whole saliva and pre-fractionated serum specimens from wild type versus dystrophic mdx-4cv mice.
Experimental features	Comparative mass spectrometry-based proteomic profiling of the saliva and serum fraction.
Data source location	Maynooth, Ireland
Data accessibility	The data are available with this article
Related research article	Murphy S, Zweyer M, Mundegar RR, Swandulla D, Ohlendieck K. Proteomic identification of elevated saliva kallikrein levels in the *mdx-4cv* mouse model of Duchenne muscular dystrophy. Biochem Biophys Rep. (2018) In press [1]

**Value of the data**

• Proteomic data presented here provide an overview of biofluid changes in the *mdx-4cv* mouse model of X-linked muscular dystrophy.
• This data provide comparative listings of proteins in saliva versus serum specimens, as well as their mass spectrometric identification.
• The mass spectrometric data are valuable to serve as a pathobiochemical biofluid signature of the dystrophin-deficient *mdx-4cv* mouse.

## 1 Data

**Fig. 1:**
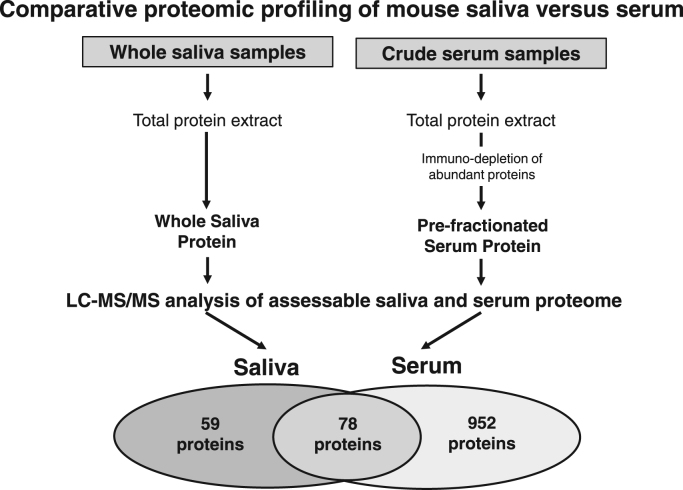
Overview of the comparative proteomic profiling of mouse saliva and serum. Shown is the flow chart of the preparation of saliva and serum protein populations for the mass spectrometry-based proteomic identification of biofluid markers. The Venn diagram illustrates the distribution of protein species between saliva and serum.

**Fig. 2:**
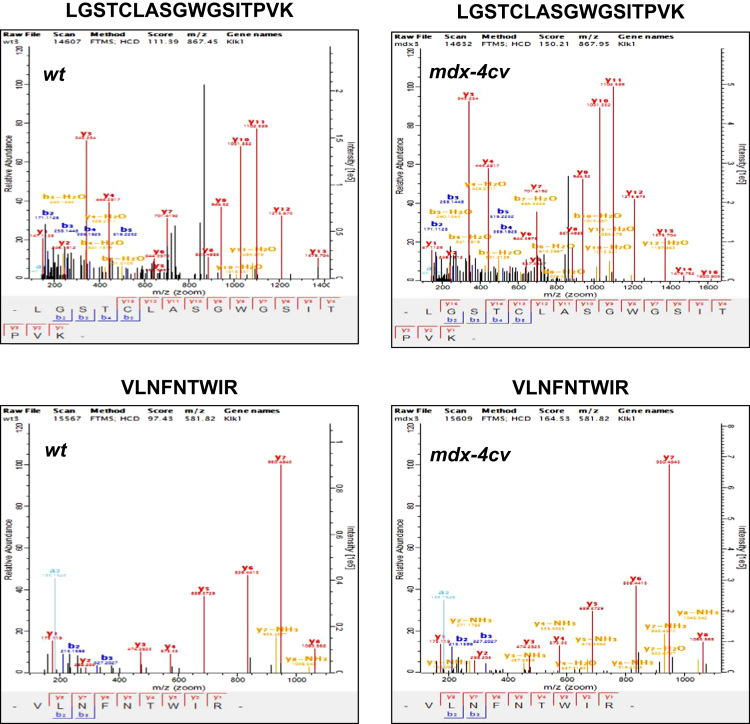
Proteomic identification of kallikrein isoform Klk1 in saliva from the wild type versus the *mdx-4cv* mouse model of Duchenne muscular dystrophy. Shown are representative MS/MS scans of the unique Klk-1 peptides LGSTCLASGWGSITPVK and VLNFNTWIR, which were identified and compared in wild type versus *mdx-4cv* saliva, respectively.

The data presented relate to the systematic survey of whole saliva using mass spectrometry-based proteomics of the *mdx-4cv* mouse model of Duchenne muscular dystrophy [1]. This accompanying article lists the proteomic identification of the total saliva protein population and the differential presence of protein species in saliva versus serum samples, as well as representative MS/MS scans of unique peptides that were used to identify the kallikrein isoform Klk-1 in wild type versus *mdx-4cv* saliva specimens. Table 1 lists the mass spectrometric profiling of the mouse saliva proteome. Listed are the protein name, gene name, the number of unique peptides, the number of total peptides, the relative molecular mass, and the estimated isoelectric point of the identified protein species. A set of typical marker proteins of whole saliva were identified, including alpha-amylase and albumin, as well as distinct isoforms of cystatin, serpin, kallikrein, cathepsin, glutathione transferase, carbonic anhydrase, mucin, pyruvate kinase, and aldolase [2], [3], [4], [5]. The identified protein species in saliva were compared with the previously established serum proteome [6]. Fig. 1 shows a Venn diagram of the distribution of proteins that are shared between saliva and serum, and protein species that are uniquely associated with saliva versus serum samples. Table 2, Table 3 list the mass spectrometric identification of proteins identified in saliva only or are shared between serum and saliva. In Table 2 are listed 59 proteins found in wild-type saliva, but not serum, including carbonic anhydrase 6, BPI fold-containing family A members 1 and 2, cystatin 10, cardiomyopathy-associated protein 5, mucin-19, and desmoplakin. Table 3 lists 78 proteins found in both serum and saliva, including alpha-amylase, cathepsin D, serum albumin, and fructose-bisphosphate aldolase A, as well as kallikrein-1 and Klk1-related peptidases b1, b3, b4, b5, b8, b9, b11, b16, b21, b22, b24, b26, and b27. In addition to the MS/MS scans of the unique peptide NNFLEDEPSAQHR shown in the accompanying research manuscript [1], Fig. 2 displays additional MS/MS scans of the unique peptides LGSTCLASGWGSITPVK and VLNFNTWIR that were used to identify the Klk-1 isoform in both wild type and mdx-4cv samples.

**Table 1:** Mass spectrometry-based proteomic identification of proteins in whole saliva from wild type mouse.

Protein name	Gene	Number of unique peptides	Number of peptides	Molecular mass kDa	Isoelectric point pI
Mucin-19	Muc19	4	4	693.1	5.54
Cardiomyopathy-associated protein 5	Cmya5	1	1	412.8	4.75
Desmoplakin	Dsp	1	1	332.7	6.80
Hornerin	Hrnr	1	1	247.4	10.33
Ovostatin	Ovos	6	6	162.2	5.26
WD repeat-containing protein 7	Wdr7	1	1	160.2	7.01
Calcium-dependent secretion activator 2	Cadps2	2	2	143.8	6.14
Pro-epidermal growth factor	Egf	10	10	133.0	6.46
Repetin	Rptn	1	1	128.5	7.61
Collagen alpha-1(I) chain	Col1a1	1	1	117.7	5.72
Lysosomal alpha-mannosidase	Man2b1	14	14	114.6	8.13
Aminopeptidase N	Anpep	4	4	109.6	5.90
Zinc finger CCHC domain-containing protein 14	Zcchc14	1	1	98.6	8.25
Dipeptidyl peptidase 4	Dpp4	1	1	87.4	6.42
Neprilysin	Mme	2	2	85.6	5.81
Heat shock protein 75 kDa, mitochondrial	Trap1	1	1	80.2	6.68
Cytosolic carboxypeptidase-like protein 5	Agbl5	1	1	80.1	8.24
Solute carrier family 15 member 1	Slc15a1	1	1	78.5	7.93
Amyloid beta A4 protein	App	1	1	78.4	4.83
Lactotransferrin	Ltf	1	1	77.8	8.53
Protein-glutamine gamma-glutamyltransferase E	Tgm3	2	2	77.3	6.81
Galactocerebrosidase	Galc	2	2	77.2	6.74
Stress-70 protein, mitochondrial	Hspa9	1	1	73.4	6.07
Keratin, type II cytoskeletal 2, epidermal	Krt2	4	5	70.9	8.06
Heat shock cognate 71 kDa protein	Hspa8	1	1	70.8	5.52
Serum albumin	Alb	3	3	68.6	6.07
Keratin, type II cytoskeletal 1	Krt1	5	7	65.6	8.15
Sulfhydryl oxidase 1	Qsox1	2	2	63.3	7.93
Keratin, type II cytoskeletal 2, oral	Krt76	8	11	62.8	8.43
Vomeromodulin	Bpifb9a	15	15	62.4	5.68
Keratin, type II cytoskeletal 5	Krt5	3	9	61.7	7.75
Prosaposin	Psap	1	1	61.4	5.19
Beta-hexosaminidase subunit beta	Hexb	9	9	61.1	8.12
Keratin, type II cytoskeletal 6B	Krt6b	1	16	60.3	8.32
Keratin, type II cytoskeletal 6A	Krt6a	2	17	59.3	7.94
Keratin, type II cytoskeletal 73	Krt73	1	3	58.9	8.09
Biotinidase	Btd	1	1	58.1	5.80
Pyruvate kinase	Pkm	3	3	57.8	7.47
N-acetylgalactosamine-6-sulfatase	Galns	1	1	57.6	6.52
Alpha-amylase 1	Amy1	10	10	57.6	6.96
Keratin, type II cytoskeletal 79	Krt79	1	3	57.5	7.69
Keratin, type I cytoskeletal 10	Krt10	9	11	57.0	5.07
Keratin, type II cytoskeletal 4	Krt4	19	21	56.2	8.15
Podocalyxin	Podxl	1	1	53.4	4.97
Aldehyde dehydrogenase family 3 member B2	Aldh3b2	2	2	52.9	6.09
Keratin, type I cytoskeletal 14	Krt14	3	7	52.8	5.17
Acidic mammalian chitinase	Chia	3	3	52.0	5.06
Angiotensinogen	Agt	1	1	52.0	5.44
Keratin, type I cytoskeletal 16	Krt16	4	7	51.6	5.20
Carboxypeptidase Q	Cpq	4	4	50.5	6.40
Aldehyde dehydrogenase, dimeric NADP-preferring	Aldh3a1	2	2	50.4	6.95
Keratin, type I cytoskeletal 42	Krt42	1	5	50.1	5.16
Elongation factor 1-alpha 1	Eef1a1	3	3	50.1	9.01
Serpin B12	Serpinb12	1	1	47.8	5.17
Keratin, type I cytoskeletal 13	Krt13	14	18	47.7	4.86
Transcobalamin-2	Tcn2	1	1	47.6	6.33
Alpha-N-acetyl-galactosaminidase	Naga	1	1	47.2	6.44
Alpha-enolase	Eno1	2	2	47.1	6.80
Rab GDP dissociation inhibitor beta	Gdi2	1	1	46.6	6.90
Chitinase-like protein 4	Chil4	4	4	44.9	6.19
Cathepsin D	Ctsd	4	4	44.9	7.15
Phosphoglycerate kinase 2	Pgk2	1	1	44.8	6.80
MANSC domain-containing protein 1	Mansc1	1	1	44.8	9.11
Renin-1	Ren1	2	2	44.3	7.17
Prostatic acid phosphatase	Acpp	1	1	43.7	6.24
Serpin B11	Serpinb11	1	1	43.5	8.94
Synaptic vesicle membrane protein VAT-1	Vat1	1	1	43.1	6.37
Serpin B6	Serpinb6	4	4	42.6	5.74
Actin, cytoplasmic 1	Actb	4	4	41.7	5.48
Adenosine deaminase	Ada	3	3	40.0	5.72
Fructose-bisphosphate aldolase A	Aldoa	2	2	39.3	8.09
Annexin A1	Anxa1	1	1	38.7	7.37
Protein LEG1	Leg1	2	2	38.3	4.36
Guanine nucleotide-binding protein subunit beta-4	Gnb4	1	1	37.4	6.16
Malate dehydrogenase, cytoplasmic	Mdh1	3	3	36.5	6.58
L-lactate dehydrogenase A chain	Ldha	2	2	36.5	7.74
Carbonic anhydrase 6	Ca6	7	7	36.5	6.60
Gamma-glutamyl hydrolase	Ggh	3	3	35.4	8.29
Polyubiquitin-B	Ubb	1	1	34.3	7.53
Triosephosphate isomerase	Tpi1	2	2	32.2	5.74
Deoxyribonuclease-1	Dnase1	3	3	32.0	4.92
Phospholipid phosphatase 1	Plpp1	1	1	31.9	7.02
Syntaxin-3	Stx3	1	1	30.9	5.63
Syntaxin-7	Stx7	3	3	29.8	5.78
Kallikrein 1-related peptidase b1	Klk1b1	4	8	29.0	8.10
Kallikrein 1-related peptidase b3	Klk1b3	4	7	29.0	6.84
Kallikrein 1-related peptidase b24	Klk1b24	3	9	28.9	8.16
Kallikrein 1-related peptidase b9	Klk1b9	5	9	28.9	7.64
Kallikrein-1	Klk1	2	6	28.8	5.12
Kallikrein 1-related peptidase b5	Klk1b5	4	7	28.7	5.59
Kallikrein 1-related peptidase b27	Klk1b27	3	9	28.7	8.56
Kallikrein 1-related peptidase b11	Klk1b11	5	9	28.7	7.14
Kallikrein 1-related peptidase b16	Klk1b16	7	9	28.7	5.64
Kallikrein 1-related peptidase b21	Klk1b21	2	8	28.7	7.37
BPI fold-containing family A member 1	Bpifa1	1	1	28.6	6.51
Kallikrein 1-related peptidase-like b4	Klk1b4	6	7	28.5	4.86
Kallikrein 1-related peptidase b8	Klk1b8	7	11	28.5	8.00
Kallikrein 1-related peptidase b26	Klk1b26	3	9	28.4	6.86
Kallikrein 1-related peptidase b22	Klk1b22	5	6	28.4	6.65
14-3-3 protein zeta/delta	Ywhaz	1	1	27.8	4.79
Cysteine-rich secretory protein 1	Crisp1	3	4	27.7	6.87
Glutathione S-transferase omega-1	Gsto1	2	2	27.5	7.36
Cysteine-rich secretory protein 3	Crisp3	1	2	27.3	8.37
Beta-nerve growth factor	Ngf	3	3	27.1	9.47
Ras-related protein Rab-27A	Rab27a	1	1	25.0	5.36
BPI fold-containing family A member 2	Bpifa2	4	4	24.7	5.01
Ras-related protein Rab-2A	Rab2a	1	1	23.5	6.54
Rho GDP-dissociation inhibitor 1	Arhgdia	1	1	23.4	5.20
Synaptosomal-associated protein 23	Snap23	1	1	23.2	4.98
Ras-related protein Rab-10	Rab10	1	3	22.5	8.38
Ras-related protein Rab-1B	Rab1b	1	3	22.2	5.73
Peroxiredoxin-1	Prdx1	1	1	22.2	8.12
Major urinary protein 3	Mup3	1	1	21.5	4.81
Vomeronasal secretory protein 2	Lcn4	1	1	21.4	5.73
Ras-related protein Rap-1A	Rap1a	1	1	21.0	6.67
Major urinary protein 5	Mup5	3	3	20.9	4.86
Placenta-expressed transcript 1 protein	Plet1	1	1	20.8	6.14
Vomeronasal secretory protein 1	Lcn3	1	1	20.6	4.60
Major urinary protein 4	Mup4	4	4	20.5	5.80
Tumor protein D52	Tpd52	1	1	20.0	4.88
Odorant-binding protein 2a	Obp2a	2	2	20.0	6.42
Odorant-binding protein 1b	Obp1b	3	3	19.4	6.29
Protein MAL2	Mal2	1	1	19.1	6.49
Destrin	Dstn	1	1	18.5	7.97
Odorant-binding protein 1a	Obp1a	4	4	18.5	5.67
Peptidyl-prolyl cis-trans isomerase A	Ppia	1	1	18.0	7.90
Nucleoside diphosphate kinase B	Nme2	3	3	17.4	7.50
Prolactin-inducible protein	Pip	4	4	16.8	4.78
Calmodulin-4	Calm4	1	1	16.8	4.89
Cystatin 10	Cst10	2	2	16.4	7.72
Superoxide dismutase [Cu-Zn]	Sod1	3	3	15.9	6.51
Submaxillary gland androgen-regulated protein 3A	Smr3a	1	1	15.5	9.09
Profilin-1	Pfn1	1	1	14.9	8.28
Protein S100-A9	S100a9	1	1	13.0	7.17
Secretoglobin family 2B member 2	Scgb2b2	1	1	12.8	5.95
Vesicle-associated membrane protein 8	Vamp8	2	2	11.4	8.19
Protein S100-A1	S100a1	1	1	10.5	4.50

**Table 2:** Mass spectrometry-based proteomic identification of proteins present in whole saliva from wild type mouse, but not serum.

**Accession Number**	**Protein name**	**Gene name**
P07744	Keratin, type II cytoskeletal 4	Krt4
Q80XI7	Vomeromodulin	Bpifb9a
P08730	Keratin, type I cytoskeletal 13	Krt13
P18761	Carbonic anhydrase 6	Ca6
Q9Z331	Keratin, type II cytoskeletal 6B	Krt6b
P07743	BPI fold-containing family A member 2	Bpifa2
Q9D3H2	Odorant-binding protein 1a	Obp1a
P11590	Major urinary protein 4	Mup4
P11591	Major urinary protein 5	Mup5
P02535-2	Isoform 2 of Keratin, type I cytoskeletal 10	Krt10
A2AEP0	Odorant-binding protein 1b	Obp1b
P61027	Ras-related protein Rab-10	Rab10
Q9JM84	Cystatin 10	Cst10
Q6UGQ3	Secretoglobin family 2B member 2	Scgb2b2
Q91Z98	Chitinase-like protein 4	Chil4
Q8C6C9	Protein LEG1 homolog	Leg1
Q91XA9	Acidic mammalian chitinase	Chia
P06281	Renin-1	Ren1
Q9JM83	Calmodulin-4	Calm4
P49183	Deoxyribonuclease-1	Dnase1
Q61900	Submaxillary gland androgen-regulated protein 3A	Smr3a
O09044	Synaptosomal-associated protein 23	Snap23
Q62472	Vomeronasal secretory protein 2	Lcn4
P38647	Stress-70 protein, mitochondrial	Hspa9
Q62471	Vomeronasal secretory protein 1	Lcn3
Q62465	Synaptic vesicle membrane protein VAT-1 homolog	Vat1
Q8BI08	Protein MAL2	Mal2
P53994	Ras-related protein Rab-2A	Rab2a
Q9ERI2	Ras-related protein Rab-27A	Rab27a
P97361	BPI fold-containing family A member 1	Bpifa1
Q61469	Phospholipid phosphatase 1	Plpp1
Q09M02–6	Isoform 6 of Cytosolic carboxypeptidase-like protein 5	Agbl5
Q62393-2	Isoform 2 of Tumor protein D52	Tpd52
Q3UU35	Ovostatin homolog	Ovos
Q8BND5-3	Isoform 3 of Sulfhydryl oxidase 1	Qsox1
Q8VIG0–2	Isoform 2 of Zinc finger CCHC domain-containing protein 14	Zcchc14
P47739	Aldehyde dehydrogenase, dimeric NADP-preferring	Aldh3a1
P10107	Annexin A1	Anxa1
E9Q3E1	Aldehyde dehydrogenase family 3 member B2	Aldh3b2
Q9R0M4	Podocalyxin	Podxl
P09041	Phosphoglycerate kinase 2	Pgk2
Q08189	Protein-glutamine gamma-glutamyltransferase E	Tgm3
P54818	Galactocerebrosidase	Galc
Q9CR33	MANSC domain-containing protein 1	Mansc1
Q9D7P9	Serpin B12	Serpinb12
Q64704-3	Isoform 3C of Syntaxin-3	Stx3
Q8CE08	Prostatic acid phosphatase	Acpp
Q920I9-2	Isoform 2 of WD repeat-containing protein 7	Wdr7
P29387	Guanine nucleotide-binding protein subunit beta-4	Gnb4
Q9QWR8	Alpha-N-acetylgalactosaminidase	Naga
Q8VHD8	Hornerin	Hrnr
Q9CQN1	Heat shock protein 75 kDa, mitochondrial	Trap1
Q8BYR5-5	Isoform 5 of Calcium-dependent secretion activator 2	Cadps2
Q9JIP7	Solute carrier family 15 member 1	Slc15a1
P12023-2	Isoform APP695 of Amyloid beta A4 protein	App
P97347	Repetin	Rptn
Q70KF4	Cardiomyopathy-associated protein 5	Cmya5
Q6PZE0	Mucin-19	Muc19
E9Q557	Desmoplakin	Dsp

**Table 3:** Mass spectrometry-based proteomic identification of proteins that are present in both saliva and serum from wild type mouse.

**Accession number**	**Protein name**	**Gene name**
P05064	Fructose-bisphosphate aldolase A	Aldoa
P00756	Kallikrein 1-related peptidase b3	Klk1b3
P07724	Serum albumin	Alb
Q01768	Nucleoside diphosphate kinase B	Nme2
P15946	Kallikrein 1-related peptidase b11	Klk1b11
P00755	Kallikrein 1-related peptidase b1	Klk1b1
P35700	Peroxiredoxin-1	Prdx1
P06151	L-lactate dehydrogenase A chain	Ldha
P15948	Kallikrein 1-related peptidase b22	Klk1b22
P07628	Kallikrein 1-related peptidase b8	Klk1b8
P15949	Kallikrein 1-related peptidase b9	Klk1b9
P17751	Triosephosphate isomerase	Tpi1
P60710	Actin, cytoplasmic 1	Actb
P52480	Pyruvate kinase PKM	Pkm
P04071	Kallikrein 1-related peptidase b16	Klk1b16
P62962	Profilin-1	Pfn1
Q9JM71	Kallikrein 1-related peptidase b27	Klk1b27
P17182	Alpha-enolase	Eno1
P36369	Kallikrein 1-related peptidase b26	Klk1b26
P08228	Superoxide dismutase [Cu-Zn]	Sod1
P14152	Malate dehydrogenase, cytoplasmic	Mdh1
P0CG49	Polyubiquitin-B	Ubb
Q61759	Kallikrein 1-related peptidase b21	Klk1b21
P17742	Peptidyl-prolyl cis-trans isomerase A	Ppia
P63017	Heat shock cognate 71 kDa protein	Hspa8
P15945	Kallikrein 1-related peptidase b5	Klk1b5
P63101	14-3-3 protein zeta/delta	Ywhaz
O88968	Transcobalamin-2	Tcn2
P00757	Kallikrein 1-related peptidase-like b4	Klk1b4
Q61754	Kallikrein 1-related peptidase b24	Klk1b24
P00687	Alpha-amylase 1	Amy1
P15947	Kallikrein-1	Klk1
Q61598-2	Isoform 2 of Rab GDP dissociation inhibitor beta	Gdi2
Q8CIF4	Biotinidase	Btd
Q99PT1	Rho GDP-dissociation inhibitor 1	Arhgdia
Q03401	Cysteine-rich secretory protein 1	Crisp1
O09131	Glutathione S-transferase omega-1	Gsto1
P04939	Major urinary protein 3	Mup3
O09159	Lysosomal alpha-mannosidase	Man2b1
P20060	Beta-hexosaminidase subunit beta	Hexb
P11859	Angiotensinogen	Agt
Q9WVJ3-2	Isoform 2 of Carboxypeptidase Q	Cpq
P10126	Elongation factor 1-alpha 1	Eef1a1
P56565	Protein S100-A1	S100a1
Q9R0P5	Destrin	Dstn
Q922U2	Keratin, type II cytoskeletal 5	Krt5
Q6IFX2	Keratin, type I cytoskeletal 42	Krt42
Q61781	Keratin, type I cytoskeletal 14	Krt14
P50446	Keratin, type II cytoskeletal 6A	Krt6a
P08071	Lactotransferrin	Ltf
P01132	Pro-epidermal growth factor	Egf
Q9Z2K1	Keratin, type I cytoskeletal 16	Krt16
Q03402	Cysteine-rich secretory protein 3	Crisp3
P01139	Beta-nerve growth factor	Ngf
Q9D1G1	Ras-related protein Rab-1B	Rab1b
P97449	Aminopeptidase N	Anpep
Q3TTY5	Keratin, type II cytoskeletal 2 epidermal	Krt2
P31725	Protein S100-A9	S100a9
Q60854	Serpin B6	Serpinb6
Q8VEN2-2	Isoform 2 of Placenta-expressed transcript 1 protein	Plet1
O70404	Vesicle-associated membrane protein 8	Vamp8
P18242	Cathepsin D	Ctsd
Q61207	Prosaposin	Psap
Q9CQV3	Serpin B11	Serpinb11
P11087-2	Isoform 2 of Collagen alpha-1(I) chain	Col1a1
P28843	Dipeptidyl peptidase 4	Dpp4
Q6NXH9	Keratin, type II cytoskeletal 73	Krt73
Q3UV17	Keratin, type II cytoskeletal 2 oral	Krt76
Q8VED5	Keratin, type II cytoskeletal 79	Krt79
P62835	Ras-related protein Rap-1A	Rap1a
P04104	Keratin, type II cytoskeletal 1	Krt1
P02816	Prolactin-inducible protein homolog	Pip
Q8K1H9	Odorant-binding protein 2a	Obp2a
Q571E4	N-acetylgalactosamine-6-sulfatase	Galns
Q9Z0L8-2	Isoform II of Gamma-glutamyl hydrolase	Ggh
P03958	Adenosine deaminase	Ada
Q61391	Neprilysin	Mme
O70439	Syntaxin-7	Stx7

## 2 Experimental design, materials, and methods

Details of the methodological approach used in this study are available in [1], [6].

### 2.1 Sample collection and processing

For the proteomic profiling of easily assessable biofluids, saliva and serum specimens were obtained from 6-month-old dystrophic *mdx-4cv* and age-matched wild type C57BL/6 mice through the Bioresource Unit of the University of Bonn [6], where mice were kept under standard conditions according to German legislation on the use of animals in experimental research. Sample collection and preparation of protein extracts were carried out as previously described in detail [1], [6]. The collected saliva and serum specimens were transported to Maynooth University on dry ice in accordance with the Department of Agriculture (animal by-product register number 2016/16 to the Department of Biology, National University of Ireland, Maynooth).

### 2.2 Mass spectrometric analysis of saliva and serum proteins

Serum samples were processed as previously described [6]. For the proteomic analysis of saliva samples, 30 µg of protein was processed by the filter-aided sample preparation (FASP) method, as described in detail by Wiśniewski et al. [7], using a trypsin to protein ratio of 1:25 (protease:protein). Following overnight digestion and elution of peptides from the spin filter, 2% trifluoroacetic acid (TFA) in 20% acetonitrile (ACN) was added to the filtrates (3:1 (v/v) dilution). Peptides were analyzed by label-free liquid chromatography mass spectrometry (LC-MS/MS) by a standardized method using an Ultimate 3000 NanoLC system (Dionex Corporation, Sunnyvale, CA, USA) coupled to a Q-Exactive mass spectrometer (Thermo Fisher Scientific) as previously described in detail [1], [6], [8], [9].

### 2.3 Protein identification and quantification

Proteins present in the wild type and the *mdx-4cv* salivary and serum proteomes were initially identified using Proteome Discoverer 1.4 against Sequest HT (SEQUEST HT algorithm, licence Thermo Scientific, registered trademark University of Washington, USA) using the UniProtKB/Swiss-Prot database, with 25,041 sequences for *Mus musculus* [1], [6]. Identified saliva peptides were then filtered using a minimum XCorr score of 1.5 for 1, 2.0 for 2, 2.25 for 3, and 2.5 for 4 charge states, with peptide probability set to high confidence. For quantitative analysis, samples were evaluated with MaxQuant software (version 1.6.1.0) and the Andromeda search engine used to explore the detected features against the UniProtKB/SwissProt database for *Mus musculus*. The following search parameters were used: (i) first search peptide tolerance of 20 ppm, (ii) main search peptide tolerance of 4.5 ppm, (iii) cysteine carbamidomethylation set as a fixed modification, (iv) methionine oxidation set as a variable modification, (v) a maximum of two missed cleavage sites, and (vi) a minimum peptide length of seven amino acids. The false discovery rate (FDR) was set to 1% for both peptides and proteins using a target-decoy approach. Relative quantification was performed using the MaxLFQ algorithm [10]. The “proteinGroups.txt” file produced by MaxQuant was further analysed in Perseus (version 1.5.1.6). Proteins that matched to the reverse database or a contaminants database or that were only identified by site were removed. The LFQ intensities were log2 transformed, and only proteins found in all eight replicates in at least one group were used for further analysis. Data imputation was performed to replace missing values with values that simulate signals from peptides with low abundance chosen from a normal distribution specified by a downshift of 1.8 times the mean standard deviation of all measured values and a width of 0.3 times this standard deviation [11]. A two-sample *t*-test was performed using *p*<0.05 on the post imputated data to identify statistically significant differentially abundant proteins.
