# Supplementary material for: Dataset on the comparative proteomic profiling of mouse saliva and serum from wild type versus the dystrophic mdx-4cv mouse model of dystrophinopathy
Source: Data Brief. 2018 Oct 27;21:1236–45. doi: 10.1016/j.dib.2018.10.082 (PMC6231363; doi:10.1016/j.dib.2018.10.082)
Supplement: Supplementary file 1 — Supplementary material [file mmc1.docx]

AUTHOR DECLARATION

Manuscript: Data in Brief [DIB-D-18-02679]

Title: Dataset on the comparative proteomic profiling of mouse saliva and serum from wild type versus the dystrophic mdx-4cv mouse model of dystrophinopathy.

Authors: Sandra Murphy^1^, Margit Zweyer^2^, Rustam R. Mundegar^2^, Dieter Swandulla^2^ and Kay Ohlendieck^1^

Affiliations: ^1^Department of Biology, Maynooth University, National University of Ireland, Maynooth, Co. Kildare, Ireland; ^2^Institute of Physiology II, University of Bonn, D‑53115 Bonn, Germany

We wish to confirm that there are no known conflicts of interest associated with this publication and there has been no significant financial support for this work that could have influenced its outcome.

We confirm that the manuscript has been read and approved by all named authors and that there are no other persons who satisfied the criteria for authorship but are not listed. We further confirm that the order of authors listed in the manuscript has been approved by all of us.

Contact email: kay.ohlendieck@mu.ie
